# Supplementary material for: Interaction between the place of residence and wealth on the risk of overweight and obesity in Bangladeshi women
Source: PLoS One. 2020 Dec 7;15(12):e0243349. doi: 10.1371/journal.pone.0243349 (PMC7721197; doi:10.1371/journal.pone.0243349)
Supplement: Attachment — Submitted filename: PLOS ONE REVIEW.docx [file pone.0243349.s001.docx]

**Manuscript: Interaction between wealth and place of residence on the risk of obesity in Bangladeshi women**

**General comments**

The manuscript can be legible for publication after addressing the comments raised. There are glaring grammatical errors. The authors need to address the comments and submit the manuscript for consideration in publication.

**Comments to the author**

**Abstract**

The title of the study indicates the focus on obesity only but from the data analysis it is clear that the authors are referring to overweight/obesity. I would suggest the title includes overweight because the authors are looking at this outcome also.

Line 28-In the conclusion section of the abstract use small ‘e’ for Education

Line 28- replace the word ‘is’ with ‘were’

There is a subsection on key messages in the abstract; I am wondering if this is consistent with PLOS One article writing format.

**Introduction**

The introduction provides background on overweight/obesity, starting at the global context and narrowing down to the Bangladeshi context. One thing I see lacking is what other studies have found on the interaction of wealth and place of residence on the risk of overweight/obesity. Are there any studies which have explored the interaction of these two socioeconomic variables? Where were these studies done? High income countries? LMICs?

Lines 59-66- The authors need to revise this section and put it in a clearer language. It is difficult to understand what the authors are saying.

Line 67, use small letter ‘o’ for the word ‘Overweight’

Line 69, remove the word ‘therefore’

**Methodology**

The methodology section is comprehensively written.

Line-116 Consider putting overweight/obesity in the sentence

Line 124- It is not clear from the statistical analyses section how the interaction between place of residence and household wealth was measured. Was it measured using the interaction effect???. Let it be clear how you analysed the interaction since this is the core of the paper.

**Results**

Line 144-145-This sentence need to be revised. It is not clear what the authors are saying

Table1- In Table 1 the totals (N) for background characteristics are not equal throughout. I expect this to be consistently the same considering the exclusion criteria which indicate that you removed all missing cases. E.g. the total for age group is **22434** but for BMI the total (N) is **22 435, and** for education it is **22432.** This is the case for all variables in the two time periods.

Line 158- correct the spelling for the word ‘overweight’

Line 163- The use of the word ‘commendable’ in the sentence is not appropriate, consider replacing it with ‘notable’. I am also not sure about ‘lower increase’ do you mean ‘small increase”???.

Please correct the title for table 2 accordingly. Thus according to what the table presents, not ‘row percentage’, e.g. Percentage of overweight/obese women by socioeconomic………………….

Under the subheading Risk factors of overweight and obesity and interaction results, the interpretation of results is not clear. It seems vague and incomprehensive. For instance, the following sentence needs revision; *Among women of different age groups, the highest overweight and obesity risk were for the 2004/07 time period women in the age group 35-39 compared with women of 15-24 years of age.*

Moreover, there are many typos in this section. The authors need to correct them. For instance in page 14, correct the following words, women for women, obesity for obesity, overweith for overweight asoocicated for associated…please correct all the typos.

In page 14, paragraph 2, the first sentence is not properly written, especially *‘associated with lessen the likelihood’*………..

The last paragraph in the result section you present results on the interaction between wealth and TV viewing, wealth and education- but in the table these results are not presented.

**Discussion**

The first paragraph is a bit disorganised and needs some organization. One would assume that you would start by indicating the study aim as you have done, and not go much into elaborating the data source and the methodology since these are covered in the respective sections. In this paragraph give indicative findings on the prevalence of overweight/obesity among women and discuss those findings. There is also need to repeat results (in percentages) in this section, as a result use words such as increasing prevalence, in which socioeconomic groups etc. The discussion section generally needs to be strengthened. Findings should clearly be discussed in the light of the study objectives. As it is, it is very difficult to deduce that from the discussion. Much of the discussion is focused on prevalence rather than the interaction between wealth and place of residence. This section also needs to be revised for the correction of typos and grammatical errors.

**References**

Please make sure that all references cited in the article appear in the reference list and follow the PLOS One referencing style.
