# Supplementary material for: Interaction between the place of residence and wealth on the risk of overweight and obesity in Bangladeshi women
Source: PLoS One. 2020 Dec 7;15(12):e0243349. doi: 10.1371/journal.pone.0243349 (PMC7721197; doi:10.1371/journal.pone.0243349)
Supplement: Attachment — Submitted filename: 05 Response to Reviewers.docx [file pone.0243349.s002.docx]

**Response to the reviewer’s comments**

| Academic Editor | | | | |
| --- | --- | --- | --- | --- |
| No. | Reviewer’s comments | Author’s response | References |  |
| 1. | Please ensure that your manuscript meets PLOS ONE's style requirements, including those for file naming. | The authors reviewed and revised the styles as per the requirement of PLOS ONE, which include reference style, file naming and other requirements as specified by the editor | N/A |  |
| 2. | a. Please provide an amended Funding Statement declaring this commercial affiliation, as well as a statement regarding the Role of Funders in your study.  b. Please also provide an updated Competing Interests Statement declaring this commercial affiliation along with any other relevant declarations relating to employment, consultancy, patents, products in development, or marketed products, etc.  c. Please include both an updated Funding Statement and Competing Interests Statement in your cover letter. We will change the online submission form on your behalf. | The funding and competing interest statements have been updated and also included in the cover letter. has been updated as follows: | Cove Letter |  |
| 3. | PLOS requires an ORCID iD for the corresponding author in Editorial Manager on papers submitted after December 6th, 2016. Please ensure that you have an ORCID iD and that it is validated in Editorial Manager. | As suggested, the corresponding author has submitted the paper with ORCID ID. |  |  |
| 4. | Please ensure that you include a title page within your main document. We do appreciate that you have a title page document uploaded as a separate file, however, as per our author guidelines we do require this to be part of the manuscript file itself and not uploaded separately.  Could you therefore please include the title page into the beginning of your manuscript file itself, listing all authors and affiliations | The title page has been included in the manuscript document. |  |  |

**Response to the reviewer’s comments**

| Reviewer#1 | | | |
| --- | --- | --- | --- |
| No. | Reviewer’s comments | Author’s response | References |
|  | This paper analyzed some specific determinants on prevalence of obesity among Bangladeshi women. Although the topic is interesting and useful in national level, the scope of the paper is reduced by a brief analysis. The analysis could bring important insights from the available data, but the Authors skipped that by summarizing the long time series data. In it's present form the paper is monotonous to attract readers and it may be benefited from some graphical representations of the results. Some of my queries and specific recommendations are given below. | Thank you so much for the rigorous feedback. As per the feedback, re-analysis was accomplished considering all the four survey periods separately to observe the trend. Furthermore, three graphical figures have been added to improve the readability of the paper. | N/A |
| 5. | There should be some statements in the rationale of the study regarding the negative long run impact of obesity, both from national and international point of view. Some references citing the statistical relation regarding the complexities of obesity among women will do. | Thank you so much for the suggestion. The introduction part has been rewritten accordingly with a sub-paragraph focusing on this issue:  “Evidence also suggests that the burden of overweight/obesity is found to be significantly higher among women than men in Bangladesh [10, 12], with negative long term consequences more in women than men [16, 17]. For instance, a study reported that the prevalence of overweight and obesity among urban and rural women in Bangladesh had been increased by 17.5% and 10.4% in the years between 1999 and 2011 [17, 18]. Moreover, another recent study conducted by Kamal et al. [14] in 2015 found that the prevalence of overweight among married women in Bangladesh is 29.2%, which is alarming.” | Please refer to the introduction part text, page no. 3-4, line 47 to 54 |
| 6. | Query: already several studies are published regarding obesity in Bangladesh and the Authors also cited some of them. What extra information are being added by this paper or how the current paper is superior over those? The rationale for focusing on these particular variables/interactions of interest are missing in the Introduction to clarify that. | Thank you so much for the suggestion. The introduction part has been rewritten accordingly with a sub-paragraph focusing on this issue:  “At the same time, only a few studies have examined the effect of an interaction term between two socio-demographic factors on overweight and obesity in women. For example, a study involving multiple middle-income countries tested the obesogenic effect of the co-existence of education and household wealth in women [11]. Further, even though urbanisation and high socio-economic status are critical determinants of overweight and obesity in adults [7, 18, 19], to our best knowledge, none have examined the effect of an interaction term between household wealth and the place of residence (urban/rural) on overweight and obesity among women in low-income countries like Bangladesh [3, 12, 15]. This lack of examination highlights the importance of investigating the effect of various socio-economic risk factors, including the interaction term between the place of residence and household wealth on overweight and obesity among women in particular [11, 19].” | Please refer to introduction part text, page no. 4, line 60 to 71 |
| 7. | Choice of the independent variables requires brief but clearer explanation regarding the physiological/behavioral factors associated with obesity. The Authors should cover-up the existing vast literature more carefully to explain the scenario of obesity among Bangladeshi women. | Thank you so much for the suggestion. A brief explanation has been provided in the measures section:  “In this study, explanatory variables were selected based on the existing literature on the socio-demographic and behavioural factors associated with obesity [8, 12, 15, 19, 24]. Age, education status or wealth index of the respondents relates to different levels of physiological and social status [12, 15, 17], while watching television indicates the sedentary behaviour of more sitting time[8]. Besides considering the literature, the significance of the chi-square tests between the primarily selected independent variables and the outcome variable (BMI category) from the bivariate analyses of BDHS data were considered to validate the rationale of choosing these variables.”  The results of the analysis were not shown in the paper. | Please refer to methodology part text, page no. 6, line 109 to 116 |
| 8. | Query: until BDHS 2014, huge proportion of the respondents used to listen radio on a regular basis. Specially during 2007~11, the new FM radio stations were popular in Bangladesh. Same concept applies for newspapers but for a more educated part of the population. Why did the Authors choose to consider only TV over mass media (by combining the joint effect of TV, Radio and newspaper together)? The Authors need to clarify this in the paper. | Thanks so much for raising the issue. In fact, we have added TV viewing as a proxy indicator of sedentary behaviour with more sitting time, prompted by the study of Ghose (2017). As our focus was on sitting time, we did not looked into the other media related exposure, which might not have related with increasing the sitting time. | N/A |
| 9. | Pooling the samples from different BDHSs reduces the scope of this paper. Instead of showing the results for two distinct decades, results from each individual survey will show the trend and it will be more useful for policy making. The Authors should present the results separately for all four surveys. | Thank you so much for raising the issue. As per your suggestion, the authors have re-analysed all the analyses and showed results for each of the four surveys, instead of showing results on pooled data of two distinct decades. It is a major change in this study and the authors were enthusiastic to perform them. Please see the relevant tables, graphs and respective descriptions in the result and discussion sections. Moreover, we have revised the text in the Material and Method section as follows:  “This study excluded participants below 15 years of age, participants with missing values for measured height and weight, and women who were pregnant at the time of the survey. Observations with missing values for the confounding variables considered in this study were also excluded from the study. After applying the exclusion criteria, the total study population from all the four periods of surveys was 54337 women aged 15-49 years.” | See Table 1, Table 2a, Table 2b and Table 3 in the manuscript. |
| 10. | Some other possible interactions in addition to the mentioned ones may have significant impact on obesity. I understand that consideration of the other interactions are outside of the scope for the current paper, however the Authors should mention some of those in the paper. | Thank you so much for the suggestion. In fact, during the analysis we considered these factors and tried to dig out the other four possible interactions, e.g., education and television viewing with wealth index and with the place of residence. As these interactions were insignificant, authors removed them from the final model. A brief explanation has been provided in the methodology part of this paper:    “As part of sensitivity analysis for the interaction effects of some other variables on overweight and obesity, this study investigated the interaction of both education and television viewing with wealth index and with the place of residence separately. The analyses found no significant interaction effects on overweight or obesity in any of the surveys of the study sample for these variables. Hence, these interactions were not included in the final models.” | Please refer to methodology part text, page no. 7, line 147 to 151 |
| 11. | The result analysis should be stronger (see the next comments). This analysis contains important time series data for obesity trend among Bangladeshi women, but the Authors did not focus on that in the findings. This applies for all three types of findings: distribution, bivariate associations, and regression analysis. | Thank you so much for raising the issues. Now, as per the comment, all the analyses have been revised to obtain the full strength. In all types of findings: distribution, bivariate associations, and regression analysis, trends of all four-time points have been captured. These are presented in all the tables and figures. | See all three tables (Table 1-3) and figures (Fig 1 – 3) |
| 12. | All the tables title need to be edited to mention the content properly. Data sources are also missing in the titles. | Thank you so much for directing the corrections. All the titles of the tables have been revised and data sources are mentioned. | See all three tables (Table 1-3) |
| 13. | What does this CI stands for in Table 1? The Authors showed the frequency in that table, not average. I suggest to remove it. | Thank you so much for directing the correction. As per the suggestion, the Confidence Intervals (CI) from table 1 have been removed. | See table 1 in page 9 |
| 14. | Table 2 is difficult to understand for weak presentation of too many information (including title). The Authors need to change the captions and stubs properly. The absolute differences should be removed. However, I highly recommend to replace this table with specific graphical representation(s). In addition, the Authors need to include appropriate graphical representation(s) to sketch the relation between the interactions and prevalence of obesity. | Thanks so much for raising the issues. As per your recommendation, two graphs have been included from the results of Table 2 to represent the prevalence of obesity over time. Titles of the three figures are as follows:  Fig. 1: Prevalence of sum of overweight and obese women aged 15-49 years over the survey periods by socio-demographic characteristics – BDHS 2004, 2007, 2011, 2014  Fig. 2: BMI status of women aged 15-49 years over the survey periods by type of residence and wealth index - BDHS 2004, 2007, 2011 and 2014  Fig. 3: BMI status of women aged 15-49 years over the survey periods by division or place of residence - BDHS 2004, 2007, 2011, 2014  However, still there remain some covariates on which bivariate tables on overweight and obesity need to be presented in the paper. Hence, we kept Table 2. As you recommended to analyse all four survey years’ data separately, we split Table 2 in two parts - Table 2a and Table 2b – with two surveys in each of these tables. This is because in one table it is impossible to present the analysis results of all the four survey years. the caption of table 2a and Table 2b have been given as follows:  “Table 2a. Prevalence of overweight and obesity by household socio-demographic characteristics in women (BDHS: 2004 and 2007)”  “Table 2b. Prevalence of overweight and obesity by household socio-demographic characteristics in women (BDHS: 2011 and 2014)” | See Table 2a and Table 2b and Fig 1 – 3 |
| 15. | In Table 3 and in it's description, the reference group should be mentioned clearly. Instead of present numeric results again in the adjacent texts, the Authors should discuss the key findings only without repeating the same numbers again. | Thank you so much for raising the problem. Now, the reference groups are mentioned in the first column of table 3 in brackets. Also in the result part, key findings are updated without repeating the same numbers. | See table 3 in page 14-15 and results in page 16 |
| 16. | The first sentence of the Discussion should be placed as the first sentence of the Conclusion. The other statements of the first paragraph of Discussion should be removed. | Thank you so much for raising the issue. As per your suggestion, the first sentence has been placed in the conclusion and the first paragraph has been re-written (see the next comment). | Please refer to Discussion part text, page no. 17-18, line 251 to 297 |
| 17. | The Discussion should focus the key findings only and it may include some useful findings. Statistical terms should be explained more carefully to avoid ambiguity (specially in page 16). Any statement addressing the policy-makers should be removed. | Thank you so much for raising the issue. The discussion has been rewritten focusing on the key findings. The statements addressing the policymakers have been removed from the discussion part and included in the conclusion part. As per your suggestion, we have rewritten the first paragraph of the discussion as below:  “The present study articulates that the prevalence of overweight and obesity among women aged 15-49 years has considerably increased from 9.96% in 2004 to 24.43 % in 2014. Using multinomial logistic regression models, the present study found that wealth was positively associated with obesity in women in all survey periods. Besides, this study found a significant multiplicative interaction between wealth and place of residence with an increased risk of obesity in 2004 and 2014 BDHS surveys. Moreover, the present study found that higher wealth, higher education, older age and increased television viewing were positively associated and the working status of women was negatively associated with overweight and obesity in women in all survey periods.” | Please refer to Discussion part text, page no. 17-18 |
| 18. | The second paragraph of page 17 should be rewritten very briefly (not more than 2/3 sentences) according to modified analysis. The modified statements should be placed in the Introduction as usefulness of the analysis. Else, the Authors should remove this paragraph. | Thank you so much for raising the issue. The second paragraph of the discussion part has been reduced and written focusing on the key findings. | Please refer to Discussion part text, page no. 17 |
| 19. | The last paragraph of the Discussion should be edited and placed in the Conclusion (check next comment). | Thank you so much for raising the issue. It has been done accordingly. | see conclusion, page 19, line 301-312 |
| 20. | Conclusion should contain a brief summary in 2/3 sentences; possible consequences in few words; limitation of the work followed by scopes of future research to overcome that; and how to extend the work. The Authors may suggest something to the policy-makers after fulfilling the structure but that's not a mandatory element. | Thank you so much for raising the issue. As per the suggestion, the conclusion part has been revised as follows:  “Overweight and obesity among women at reproductive age is a growing public health concern in Bangladesh. This study offered insights that urban wealthier women of reproductive age are more prone to be obese. However, the underlying reasons for the greater risk of urban wealthier women to be obese require further investigation. The present study has some limitations: first, the present study is unable to draw directional causal inferences as it is based on a cross-sectional design. Additionally, this study cannot incorporate some critical risk factors of obesity, such as respondents' dietary habits, physical activity, sedentary behaviour, and comorbid conditions, due to their unavailability in BDHS data sets. Future studies may recheck the association following prospective longitudinal research design where individuals will have been tracked and thus can capture the within-person change in BMI. . Finally, we suggest that policymakers should undertake intervention programs targeting urban wealthier women who are more prone to be obese.” | see conclusion part page 19, line 301-312 |
| 21. | The manuscript requires a careful edit for small typos and consistencies in citation style. | Thank you so much for raising the issue. As per the suggestion, all the references have been checked and edited where necessary. | N/A |

**Response to the reviewer’s comments**

| Reviewer#2 | | | |
| --- | --- | --- | --- |
| No. | Reviewer’s comments | Author’s response | References |
|  | The manuscript can be legible for publication after addressing the comments raised. There are glaring grammatical errors. The authors need to address the comments and submit the manuscript for consideration in publication. | The authors express their gratitude to the reviewer for the time and effort. All the comments we received on this paper have been taken into account to improve the quality of the article, and we present our reply to each of them separately below. Furthermore, intensive proofreading and editing have been performed to eliminate grammatical errors. | Not Applicable (N/A) |
| 1. | **Abstract**  The title of the study indicates the focus on obesity only but from the data analysis it is clear that the authors are referring to overweight/obesity. I would suggest the title includes overweight because the authors are looking at this outcome also. | Thank you so much for the suggestion. Considering your feedback, the new title has been given as follows.  “Interaction between the place of residence and wealth on the risk of overweight and obesity in Bangladeshi Women” | Please see the revised title in page 1. |
| 2. | Line 28-In the conclusion section of the abstract use small ‘e’ for Education | Thank you so much for the correction. As the sentence has been removed, the problem is now eliminated. | N/A |
| 3. | Line 28- replace the word ‘is’ with ‘were’ | Thank you so much for the correction. As the sentence has been removed, the problem is now eliminated. | N/A |
| 4. | There is a subsection on key messages in the abstract; I am wondering if this is consistent with PLOS One article writing format. | Thank you so much for raising this issue. As this section is redundant as per the requirement of this journal, it has been removed. | N/A |
| 5. | **Introduction**  The introduction provides background on overweight/obesity, starting at the global context and narrowing down to the Bangladeshi context. One thing I see lacking is what other studies have found on the interaction of wealth and place of residence on the risk of overweight/obesity. Are there any studies which have explored the interaction of these two socioeconomic variables? Where were these studies done? High income countries? LMICs? | Thank you so much for raising the issue. The introduction part has been revised and rewritten to address all of your comments. | Please refer to the Introduction section, page 3-4, lines 28 to 75. |
| 6. | Lines 59-66- The authors need to revise this section and put it in a clearer language. It is difficult to understand what the authors are saying. | Thank you so much for raising the issue. This section has been revised as follows:  “Worldwide, many studies have investigated the correlates of overweight and obesity among children and adults; however, little is known concerning women living in low-income countries [5, 17, 18]. The majority of previous studies conducted in developing countries, including Bangladesh, have focused on the prevalence of overweight and obesity in women, and tested to what extent a single socio-economic, reproductive or lifestyle factor influences women to be overweight or obese [1, 2, 5, 14]. At the same time, only a few studies have examined the effect of an interaction term between two socio-demographic factors on overweight and obesity in women.” | Please refer to the Introduction section, page 4, lines 55 to 62. |
| 7. | Line 67, use small letter ‘o’ for the word ‘Overweight’ | Thanks for locating the error. Now the section has been re-written. | N/A |
| 8. | Line 69, remove the word ‘therefore’ | Thanks for the suggestion. The word ‘therefore’ has been removed. | Please see the text in the introduction section, page 4, line 72-75 |
| 9. | **Methodology**  The methodology section is comprehensively written. | Thank you so much for the remarks. This has been further improved considering the subsequent feedback. | N/A |
| 10. | Line-116 Consider putting overweight/obesity in the sentence | Thank you so much for raising the issue. The changes have been made accordingly. | Please see page 6, line 125 |
| 11. | Line 124- It is not clear from the statistical analyses section how the interaction between place of residence and household wealth was measured. Was it measured using the interaction effect???. Let it be clear how you analysed the interaction since this is the core of the paper. | Thank you so much for your suggestion. The methodical description of measuring interaction effect has been included in the text:  “To examine the central hypothesis of whether the place of residence modified the association between wealth and overweight/obesity, interaction terms between the place of residence and wealth index were fitted along with the main effects to measure the interaction effect for overweight and obesity in the logistic regression models for all the survey periods separately. However, during the analyses, the regression models were controlled with the relevant socio-demographic characteristics (educational status, watching television, current working status and use of contraceptive method) and all the results were reported with adjusted risk ratios (ARR) and 95% confidence interval. Before fitting the models, the potential collinearity of the predictor variables and the outcome variable were examined using the variance inflation factors.” | Please see the text in the Method section, page 7, line 137-146 |
| 12. | **Results**  Line 144-145-This sentence need to be revised. It is not clear what the authors are saying | Thank you so much for the query. The text has been changed as follows to dispel the ambiguity:  “The trend in the prevalence revealed a two-fold increase of both overweight and obesity in women over the study period, 2004-2014.” | Please see the text in the results section, page 7, line 165-166. |
| 13. | Table1- In Table 1 the totals (N) for background characteristics are not equal throughout. I expect this to be consistently the same considering the exclusion criteria which indicate that you removed all missing cases. E.g. the total for age group is **22434** but for BMI the total (N) is **22 435, and** for education it is **22432.** This is the case for all variables in the two time periods. | Thanks so much for locating the errors. As per your suggestion, we have streamlined the exclusion criteria and now, across all the variables, the total number of observations is 54337 with four years breakdown as follows: 10612 in 2004, 10267 in 2007, 16678 in 2011 and 16780 in 2014. Also, note that we have included all four surveys separately in all levels of the analyses as per the feedback of the reviewer#1. Hence, we have revised the text in the Material and Method section as follows:  “This study excluded participants below 15 years of age, participants with missing values for measured height and weight, and women who were pregnant at the time of the survey. Observations with missing values for the confounding variables considered in this study were also excluded from the study. After applying the exclusion criteria, the total study population from all the four periods of surveys was 54337 women aged 15-49 years.” | Please see the text in the Method section, page 5, line 89-100 and also the Table 1 from page 9, line 173 onward. |
| 14. | Line 158- correct the spelling for the word ‘overweight’ | Thank you so much for raising the issue. Corrected accordingly. | Please see the text in the page 8, line 163 |
| 15. | Line 163- The use of the word ‘commendable’ in the sentence is not appropriate, consider replacing it with ‘notable’. I am also not sure about ‘lower increase’ do you mean ‘small increase” ??? | Thanks for raising the issues. Yes, I meant small increase and the text has been corrected referring to Fig 1, as per the feedback of another reviewer:  “There was a notable small increase in those who were in the age group of 15 to 24 years, have no children, never watch television and used hormonal contraceptive methods (see Fig. 1).” | Please see the text in page 10, line 181-183 |
| 16. | Please correct the title for table 2 accordingly. Thus according to what the table presents, not ‘row percentage’, e.g. Percentage of overweight/obese women by socioeconomic…………………. | Thank you so much for directing the corrections. Now, it has been divided into two tables and the titles have been revised as follows:  Table 2a. Prevalence of overweight and obesity by household socio-demographic characteristics in women (BDHS: 2004 and 2007)  Table 2b. Prevalence of overweight and obesity by household socio-demographic characteristics in women (BDHS: 2011 and 2014) | N/A |
| 17. | Under the subheading Risk factors of overweight and obesity and interaction results, the interpretation of results is not clear. It seems vague and incomprehensive. For instance, the following sentence needs revision; *Among women of different age groups, the highest overweight and obesity risk were for the 2004/07 time period women in the age group 35-39 compared with women of 15-24 years of age.* | Thank you so much for raising the issue. The text has been changed in this section as follows:  “In 2004, the women in the 35-49 age group were 4.64 times and 3.35 times more likely to be overweight and obese respectively concerning the women aged 15-24 years. In 2014, women of this older age group [35-49] were 2.82 times and 3.71 times more likely to be overweight and obese respectively, compared to the young age [15-24] women. Thus, there was a consistent trend of women being more likely to be overweight and obese in all the four survey periods over the decades for the age groups of 25-34 years and 35-49 years, with higher odds for the latter age group (see Table 3).” | Please see the result section, page 13, line 217-223 |
| 18. | Moreover, there are many typos in this section. The authors need to correct them. For instance in page 14, correct the following words, women for women, obesity for obesity, overweight for overweight associcated for associated…please correct all the typos. | Thank you so much for the comment. All the typos have been corrected. Furthermore, a copy-editing has been done by a professional of USQ (see the acknowledgement) to make the manuscript more readable. | N/A |
| 19. | In page 14, paragraph 2, the first sentence is not properly written, especially *‘associated with lessen the likelihood’*……….. | Thank you so much for your suggestion. The sentence has been reviewed as follows:  “Working women were less likely to be overweight and obese in all three survey time points, except for the 2011 survey year which did not show any association in this regard.” | Please refer to the results section, page 16, line 240-241. |
| 20. | The last paragraph in the result section you present results on the interaction between wealth and TV viewing, wealth and education- but in the table these results are not presented. | Thank you so much for raising the issue. As these analyses were not included in the final model, we have removed them from the results and added a clarification in the methodology section.  The method section has been revised as follows:  “As part of sensitivity analysis for the interaction effects of some other variables on overweight and obesity, this study investigated the interaction of both education and television viewing with wealth index and with the type of residence separately. The analyses found no significant interaction effects on overweight or obesity in any of the surveys of the study sample for these variables. Hence, these interactions were not included in the final models.” | Please refer to the methodology section, page 7, lines 147-151. |
| 21. | **Discussion**  The first paragraph is a bit disorganised and needs some organization. One would assume that you would start by indicating the study aim as you have done, and not go much into elaborating the data source and the methodology since these are covered in the respective sections. In this paragraph give indicative findings on the prevalence of overweight/obesity among women and discuss those findings. There is also need to repeat results (in percentages) in this section, as a result use words such as increasing prevalence, in which socioeconomic groups etc. The discussion section generally needs to be strengthened. Findings should clearly be discussed in the light of the study objectives. As it is, it is very difficult to deduce that from the discussion. Much of the discussion is focused on prevalence rather than the interaction between wealth and place of residence. This section also needs to be revised for the correction of typos and grammatical errors. | Thank you so much for raising the issue. The discussion part has been rewritten with the utmost effort to eliminate the problems as per this note. | Please refer to the discussion part text, page no. 17-18, line 250 to 297 |
| 22. | **References**  Please make sure that all references cited in the article appear in the reference list and follow the PLOS One referencing style. | Thank you so much for the suggestion. The authors have checked all the references to ensure the compliance of PLOS One referencing style. | N/A |
